# Investigating the causal risk factors for self-harm by integrating Mendelian randomisation within twin modelling

**DOI:** 10.1007/s10519-022-10114-x

**Published:** 2022-09-14

**Authors:** Kai Xiang Lim, Olakunle Ayokunmi Oginni, Kaili Rimfeld, Jean-Baptiste Pingault, Frühling Rijsdijk

**Affiliations:** 1 Social, Genetic and Developmental Psychiatry Centre, Institute of Psychiatry, Psychology and Neuroscience, King’s College London, London, UK; 2 Department of Psychology, Royal Holloway University of London, London, UK; 3 Department of Clinical, Educational and Health Psychology, Division of Psychology and Language Sciences, University College London, London, UK; 4 Faculty of Social Sciences, Anton de Kom University of Suriname, Paramaribo, Suriname

**Keywords:** Mental health, Mendelian randomisation, Direction of Causation, MR-DoC, suicidal self-harm, non-suicidal self-harm

## Abstract

Previous genetically informed studies have uncovered likely causal relationships between mental health problems and self-harm but resulting causal estimates may be biased due to unmediated pleiotropy. By fitting Mendelian Randomization - Direction of Causation (MR-DoC) models that explicitly model pleiotropy, we investigated the effect of four quantitatively measured mental health problems - major depressive disorder (MDD), schizophrenia, attention-deficit hyperactivity disorder (ADHD), and insomnia, on non-suicidal self-harm (NSSH) and suicidal self-harm (SSH), separately. We used data of 12,723 twins (56.6% females) in the Twins Early Development Study. Besides substantial pleiotropy, we found effects from child-rated depressive symptoms to both NSSH (*β* = 0.194, 95% CIs: 0.131, 0.257) and SSH (*β* = 0.210, 95% CIs: 0.125, 0.295). Similarly, effects flowed from parent-rated depressive symptoms to NSSH (*β* = 0.092, 95% CIs: 0.004, 0.181) and SSH (*β* = 0.165, 95% CIs: 0.051, 0.281). We did not find evidence of aetiological difference between NSSH and SSH.

**Supplementary Information:**

The online version contains supplementary material available at 10.1007/s10519-022-10114-x.

## Introduction

Every year, more than 700,000 people lose their lives due to suicide, which is the fourth leading cause of mortality among young people after road injury, tuberculosis and interpersonal violence (World Health Organisation, 2021). Among many risk factors, one of the strongest predictors for suicide death is prior history of self-harm (Franklin et al. 2017), which can be defined as any act of self-injury or self-poisoning carried out by an individual regardless of intention or motivation (Hawton et al. 2003). Self-harm can also be further delineated into suicidal self-harm (SSH) and non-suicidal self-harm (NSSH), i.e., with and without suicidal intent. The fifth edition of the Diagnostic and Statistical Manual of Mental Disorders (DSM-5) has proposed self-harm to be divided along the dimension of suicidal intent into two separate conditions for further study, i.e. non-suicidal self-injury and suicidal behaviour disorder (American Psychiatric Association 2013).

Genetically informed studies have offered some insights into the aetiology of self-harm. Using the classical twin design, Lim et al. (2021) found strong phenotypic (*r* = 0.87) and genetic (*r*_*g*_ = 0.94) correlations between NSSH and SSH; and that the genetic and environmental relationships of 17 mental health measures with NSSH were comparable to those with SSH. In that twin study, major depressive disorder (MDD) symptoms and insomnia were identified to have the strongest phenotypic and genetic correlations with both NSSH and SSH among 17 mental health measures (Lim et al. 2021). This was also supported by two recent molecular genetic studies, where MDD and insomnia had significant moderate genetic correlations with self-harm thoughts and behaviours (Campos et al. 2020; Ruderfer et al. 2019). The evidence suggests that insomnia and MDD may be potential causal risk factors for self-harm given their higher phenotypic and genetic correlations. However, a causal relationship cannot be inferred merely from their correlations.

Mendelian randomisation is an instrumental variable approach which uses genetic data to infer causality (Burgess et al. 2015). Genetic variants robustly associated with an exposure are used as proxies to test its effect on an outcome. Using MR, a UK Biobank study found that MDD, schizophrenia and attention-deficit/hyperactivity disorder (ADHD) were the most plausible causal risk factors for self-harm among 24 possible individual risk factors, such as mental health problems, substance use, cognitive traits, personality traits, and physical traits (Lim et al. 2020). Another study using MR found no evidence of a causal effect of smoking on suicide attempt (Harrison et al. 2020). Cannabis use was found to have a possible causal effect on suicide attempt, when there was no control for other mental health problems in a multivariable MR model (Orri et al. 2020). In summary, previous genetically informed designs identified insomnia as a potential causal risk factor for self-harm due to its higher genetic correlation with self-harm, whereas schizophrenia, ADHD, and MDD are the more plausible causal risk factors for self-harm as tested by MR.

However, genetic correlation can emerge in the absence of causality, where there is unmediated pleiotropy. Unmediated pleiotropy exists when a genetic instrument for the exposure leads to the outcome through a different pathway, rather than via the exposure (mediated pleiotropy). The MR design, on the other hand, relies on some assumptions that can be easily violated. For example, it assumes that the genetic variants used are not associated with the outcome through unmediated pleiotropic pathways (Davey Smith and Hemani 2014). Although there are MR sensitivity analyses which can be used to account for possible violations of this assumption, they are often less powerful in detecting causal effects, and the presence of unmediated pleiotropy remains possible, which can lead to false conclusions about causal relationships by biasing the magnitude of effect (Minică et al. 2018).

To address this issue, a newly developed method, the Mendelian Randomisation – Direction of Causation (MR-DoC) model, can be implemented when molecular genetic data from monozygotic (MZ) and dizygotic (DZ) twin pairs are available (Minică et al. 2018). The MR-DoC model integrates both Mendelian randomisation (MR) and the direction of causation (DoC) twin model to further strengthen causal inference. It allows some relaxation of the MR assumptions by controlling for unmediated pleiotropy and can potentially increase the statistical power relative to standard MR approaches; however, it requires other sets of assumptions (Minică et al. 2018). A more detailed description of the MR-DoC model can be found in the Methods section.

There is an ongoing debate about whether NSSH and SSH should be considered as two separate entities or are on the same continuum (Kapur et al. 2013). Although they are highly correlated (Klonsky et al. 2013; Nock et al. 2006), they are also distinctive in terms of intention, frequency, prevalence, attitudes towards life and death, and lethality of methods (Muehlenkamp and Gutierrez 2007). In a UK adolescent sample, it was found that some risk factors appear to be specific to NSSH (higher IQ and maternal education) and SSH (lower IQ and parental self-harm), although these two types of self-harm also share some common risk factors (Mars et al. 2014). A Norwegian study also found that satisfaction with social support is a protective factor for NSSH whilst attachment to parents is a protective factor for SSH, although there are also risk and protective factors common to NSSH and SSH (Wichstrøm 2009). Given that the MR-DoC causal effects may be potentially different for NSSH and SSH, in this study we investigated the causal effects for NSSH and SSH separately.

The present study investigated the causal effects for self-harm with two aims. The primary aim was to investigate the causal relationships from the aforementioned mental health problems (i.e., MDD, ADHD, schizophrenia and insomnia) to self-harm using the MR-DoC design. The secondary aim was to investigate possible aetiological differences between NSSH and SSH by investigating their causal relationships with mental health problems separately.

## Methods

This project was pre-registered on the Open Science Framework prior to accessing the data: https://osf.io/284af.

### Participants

Participants were from the Twins Early Development Study (TEDS), a longitudinal study which follows a population-based sample of twins who were born between 1994 and 1996 in England and Wales. After birth, their parents were contacted to join the study and the families were followed-up across development. At the beginning of data collection, the sample characteristics were comparable to that of the UK census data, and have remained reasonably representative for their birth cohort despite some attrition (Rimfeld et al. 2019). Across development, data were collected in multiple waves using combinations of posted questionnaires and test booklets, telephone, web-based platforms, and at age 21 years, a smartphone app. More details about the recruitment process and the sample are described in Rimfeld et al. (2019).

**Fig. 1:**
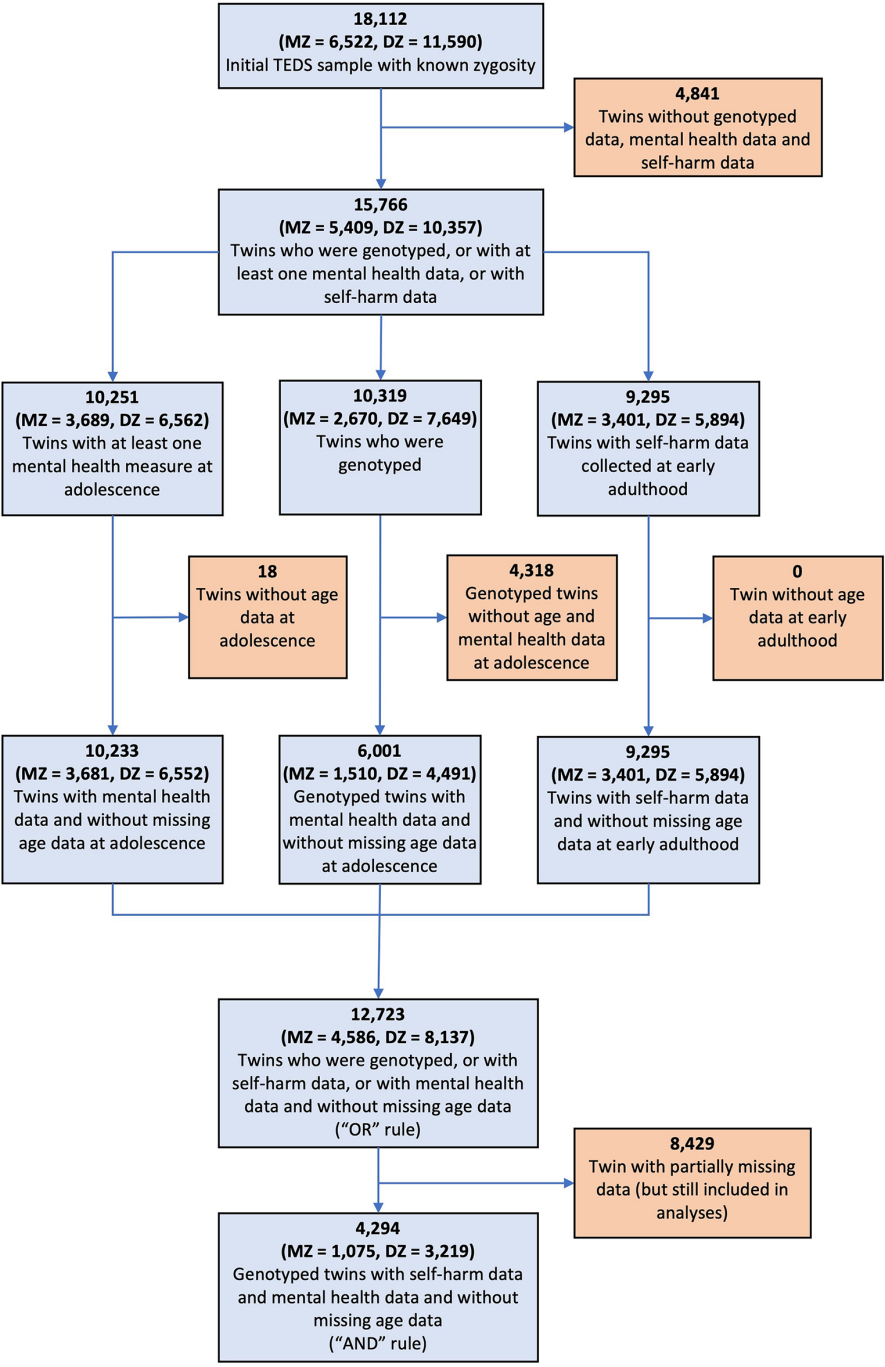
Flowchart showing the number of twins in the analyses. As OpenMx uses full information maximum likelihood to handle missing data, data from twins with partially missing data (e.g. genotyped twins who do not necessarily have self-harm data) were used in the analyses. We showed the number of twins with missing age data as age was a covariate used in the analyses. Other covariates (e.g., sex) did not have missing data

In the current study, there were a total of 12,723 twins (56.6% females), from which 10,233 twins had at least one mental health data, 6,001 twins were genotyped, and 9,295 twins had self-harm data (see Fig. 1).

### Self-harm measures (outcome variables)

Data for self-harm were collected during early adulthood (mean age = 22.3 years, *SD* = 0.92 years). The self-harm questions used in this study were taken and adapted from the Child & Adolescent Self-harm in Europe (CASE) study (Madge et al., 2008). Participants were first asked, ‘In your lifetime, have you ever hurt yourself on purpose in any way (e.g., by taking an overdose of pills, or by cutting yourself?)’. Participants were given five options to respond, ranging from ‘No’, ‘Yes, once or twice’, ‘Yes, 3–5 times’, ‘Yes, 6–10 times’ to ‘Yes, more than 10 times’.

If the participants answered ‘Yes’, they were then asked to answer two follow-up questions related to NSSH and SSH. For NSSH, participants were asked, ‘In your lifetime, have you ever hurt yourself on purpose without intending to kill yourself?’. For SSH, participants were asked, ‘In your lifetime, on any of the occasions you have hurt yourself on purpose, have you ever seriously wanted to kill yourself?’. The options available for these two questions were the same as the first question. Participants who did not endorse a history of self-harm in the first question were coded as ‘No’ in the subsequent NSSH and SSH questions.

### Mental health measures (exposure variables)

Four mental health measures collected in adolescence (mean age = 16.3 years, SD = 0.69 years) were used in the current study. These mental health measures correspond to the four mental health problems stated in the study background, i.e., MDD, ADHD, schizophrenia and insomnia:

1. For depressive symptoms, we used the Moods and Feelings Questionnaire (MFQ; Angold et al. 1995), which is an 11-item questionnaire for rating depressive symptoms in epidemiological studies among children and adolescents, rated on a 3-point Likert scale ranging from 0 to 2. These item responses were summed and used in subsequent analyses with higher scores indicating higher depressive symptoms. There were child-rated and parent-rated MFQs, and the Cronbach’s alphas were 0.88 and 0.86 respectively. In current study, the child-rated and parent-rated MFQ measures are correlated at *r* = 0.38, *p* < 0.001.
2. For ADHD symptoms, we used Conner’s Parent Rating Scale (CPRS; Conners, 2001), which is an 18-item DSM-IV ADHD symptom measure, rated by parents on a 4-point Likert scale ranging from 0 to 3. Higher summed ratings indicate higher ADHD symptoms. Cronbach’s alpha was 0.90.
3. For symptoms related to schizophrenia, we used the Specific Psychotic Experiences Questionnaire (SPEQ; Ronald et al. 2014), which is a questionnaire with six subscales used to measure psychotic experiences. Five subscales were child-rated (hallucinations [Cronbach’s alpha = 0.88], paranoia [Cronbach’s alpha = 0.93], grandiosity [Cronbach’s alpha = 0.86], anhedonia [Cronbach’s alpha = 0.77], cognitive disorganisation [Cronbach’s alpha = 0.77]) and one subscale was parent-rated (negative symptoms, Cronbach’s alpha = 0.86). Hallucination, paranoia and anhedonia subscales were rated on 6-point Likert scales ranging from 0 to 5. Grandiosity and negative symptoms were rated on 4-point Likert scales ranging from 0 to 3. Cognitive disorganisation was rated on a binary “yes/no” scale (scored 1 and 0 respectively). Higher summed ratings indicate higher measured traits. These subscales were adapted from pre-existing measures for adults to assess psychotic experiences for adolescents. They measure different facets of psychotic experiences in adolescence and are always analysed individually.
4. For insomnia, we used the child-rated Insomnia Severity Index (Bastien et al. 2001), which is a 7-item instrument used to quantify perceived insomnia severity. Four items were rated on a 5-point Likert scale ranging from 0 to 4 and three items were rated on a 6-point Likert scale ranging from 0 to 5. Higher summed ratings indicate more severe insomnia symptoms. Cronbach’s alpha was 0.89.

### Genotypic data and polygenic risk scores (instrumental variables)

A subsample of 6,001 twins from TEDS who were genotyped and passed quality control are included in this study (see Fig. 1). This consisted of 1,510 MZ twin individuals (i.e., only one twin from each MZ twin pair) and 4,491 DZ twins individuals (including 1,992 complete twin pairs). Only one individual per MZ twin pair was genotyped as MZ twins are almost genetically identical. Details of genotyping and quality control can be found elsewhere (Selzam et al. 2019). Polygenic risk scores (PRS) were computed by the TEDS team using the software LDPred (Vilhjálmsson et al. 2015). A prior on the fraction of causal markers of 1 was applied in generating the PRS, assuming an infinitesimal model where all genetic markers are involved in trait development. We used publicly available summary statistics from genome-wide association studies (GWAS) where TEDS is not part of, such that there is no overlap between the GWAS sample and TEDS sample. Using these summary statistics, PRS for the following were generated:

1. PRS for ADHD, derived from a GWAS conducted by the Psychiatric Genomics Consortium (PGC; Demontis et al. 2019).
2. PRS for MDD, derived from the PGC GWAS for MDD (excluding participants from 23&Me; Wray et al. 2018).
3. PRS for schizophrenia, derived from the PGC GWAS for schizophrenia (Pardiñas et al. 2018).
4. PRS for insomnia, derived from a GWAS for insomnia symptoms in the UK Biobank (Hammerschlag et al. 2017).

### Statistical analyses

The statistical analyses were conducted in two stages in the R version 3.6.2 environment (R Core Team 2017). In the first stage, we carried out PRS regression analyses to ensure the PRS fulfil the first assumption in standard MR as instrumental variables i.e., association with the exposure variable. The PRS that fulfil the assumption were then used in the second step of our statistical analysis, which is to build MR-DoC models using structural equation modelling.

### PRS regression analyses

We first tested the association between PRS and their respective mental health measures in regression analyses. The first 10 principal components (PCs), chip plates used, batches, sex and age were included as covariates in these regression models (In MR-DoC models, these covariates were adjusted for by residualising the PRS). We used the gee package in R and controlled for non-independence of the twin structure using the “exchangeable” argument in the gee() function, which estimates intra-class twin correlation (Halekoh et al. 2006). Robust standard errors were estimated. To control for multiple testing, we applied the false discovery rate (FDR) correction for the *p*-values obtained from the regression analyses, with a threshold of *q* < 0.05 to define significance.

### MR-DoC Models

PRS that were significantly associated with their respective mental health measures were then selected as instrumental variables for the MR-DoC models.

**Fig. 2:**
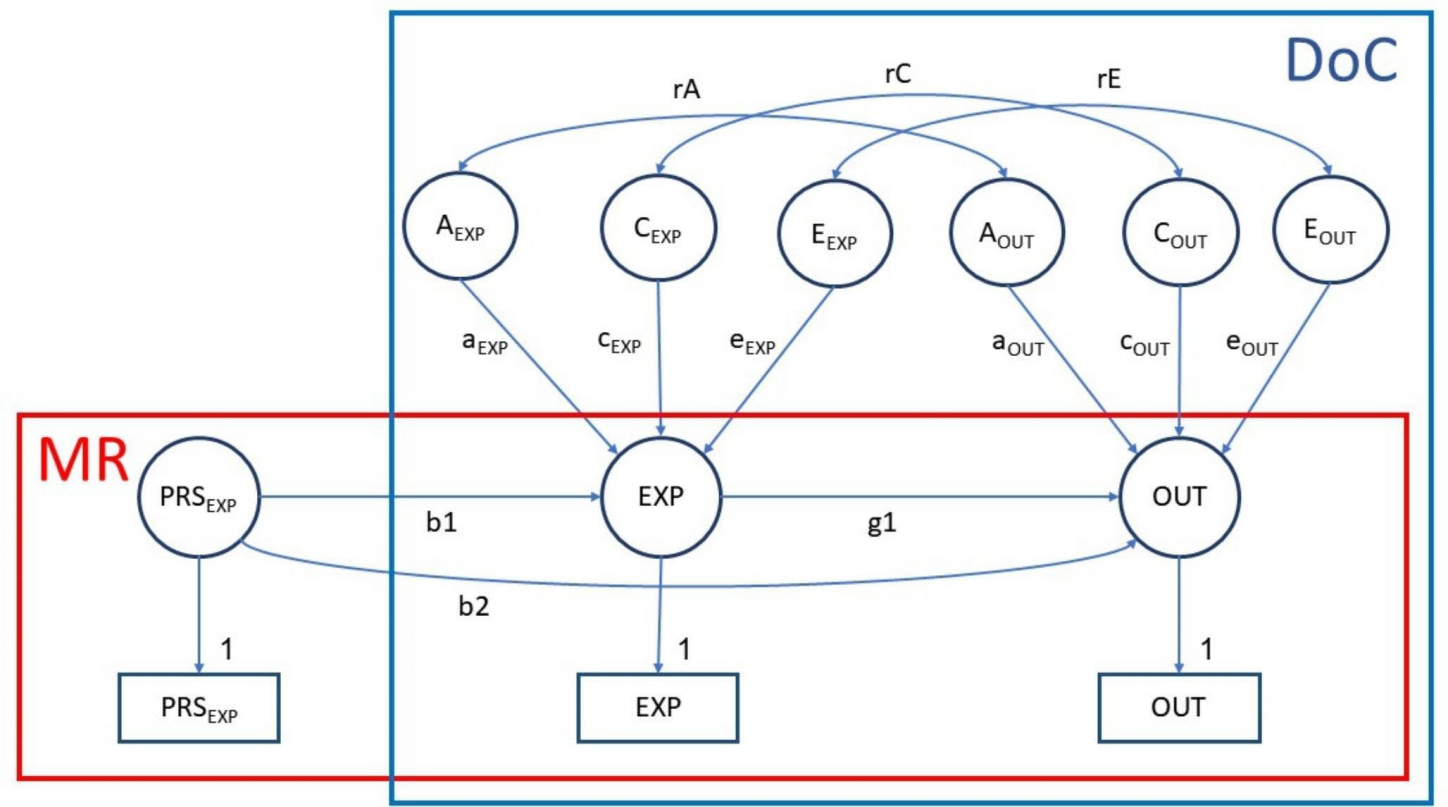
Illustration of the MR-DoC model. The red box corresponds to the MR part of the model, whereas the blue box corresponds to the DoC part of the model. Path b1 indicates the association between the instrument (PRS_EXP_ - polygenic risk score of the exposure variable) and its corresponding exposure trait (EXP). Path b2 indicates unmediated pleiotropy i.e., the direct path from PRS_EXP_ to the outcome (OUT) variable. Path g1 is the causal path between EXP and OUT. A, C and E refer to latent additive genetic, shared environmental and non-shared environmental influences on EXP and OUT, whereas rA, rC and rE are the correlation coefficients between the genetic, shared, and non-shared environmental influences on the exposure and the outcome variables. Note that this model is under-identified due to the inclusion of the unmediated pleiotropic (b2) path. In current study, rC was zero as there was no C_OUT_ influence, and rE was constrained to zero to improve resolution of the model (see explanations below)

The MR-DoC model uses PRS and twin data, combining the strengths of Mendelian randomization (MR) and the Direction of Causation (DoC) model (See Fig. 2; Minică et al. 2018). By leveraging the robust findings from GWAS, MR uses genetic variants as instrumental variables to detect the causal effect of a hypothesised, modifiable exposure (e.g., risk of a mental health problem) on an outcome (e.g., NSSH or SSH; Davey Smith and Hemani 2014). MR relies on three main assumptions, the instruments should be: (i) robustly associated with the exposure variable (i.e., b1 in Fig. 2 is significant); (ii) if associated with the outcome, this association is only through the exposure (i.e., no unmediated pleiotropy, or no b2 in Fig. 2); and (iii) independent of confounders of the exposure-outcome association. Although PRS often predict substantially more variance in exposures than all genome-wide significant genetic variants put together, which is an advantage in instrumental variable analysis, they are prone to unmediated pleiotropy.

The classic DoC model, on the other hand, uses cross-sectional twin data to test reciprocal causal relationships between two traits by leveraging differential genetic and environmental influences on two variables of interest (e.g., A and E influences on depressive symptoms versus A, C and E influences on NSSH; Heath et al. 1993). It can test whether the association between two traits is best explained by (i) unidirectional causal effects in either direction, (ii) simultaneous bidirectional causal effects, or (iii) common A, C or E influences.

By integrating MR and DoC models, the causal effect (g1 path) can be estimated (using cross-sectional twin data like in a classic DoC model). Simultaneously, the unmediated pleiotropy (i.e., b2 path) is also estimated by specifying PRS as the instrumental variable. This allows causation to be detected even in the presence of unmediated pleiotropy (Minică et al. 2018). As MZ twins share 100% of their segregated genes and DZ twins share on average 50% of their segregated genes, we were able to decompose the variance of the traits into genetic and environmental components. The additive genetic influences (A) assume allelic effects are additive rather than interactive. The shared environmental influences (C) are environmental factors shared by the twins that make them more similar to each other, whereas non-shared environmental influences (E) are environmental factors which are unique to each twin and also measurement errors. Within the DoC model, the genetic and environmental correlations between the two traits investigated can also be estimated from the cross-twin cross-trait correlations.

The full model (as specified in Fig. 2) is under-identified due to the inclusion of the causal (g1) path along the correlation paths rA, rC and rE. However, all parameters can be identified if the exposure and outcome variables have different variance component influences e.g., ACE influences on the exposure and AE influences on the outcome. By estimating only C_x_, rC and Cy are constrained to zero by default (see Minică et al., [2018], Scenario 6 in Table 1). Based on earlier research using the same sample that showed that depression and ADHD are ACE traits (Greven et al. 2011; Waszczuk et al. 2015, 2021) whereas NSSH and SSH are AE traits (Lim et al. 2021), we initially adopted ACE models for the exposures and AE models for the outcomes, with rC and Cy constrained to zero by default. However, this model had a low resolution (i.e., the confidence intervals were extremely wide e.g., -37 to 477 for a causal path estimate of 0.66). We therefore opted to further constrain rE to 0 as this is commonly done in MZ twin difference models (e.g., Kohler et al. 2011; Lim et al. 2018) to improve resolution. All ACE components of the exposures and AE components of the outcomes in the final models were significant (see Table S1). As sensitivity checks (Minică et al. 2018), we investigated the effect of fixing rE to 0 by fixing rE to different values and inspecting changes in the estimates of the causal paths (g1) in each of the MR-DoC models and comparing the goodness of fit using Akaike Information Criterion (AIC).

**Table 1:** Descriptive statistics for the prevalence of NSSH and SSH

Self-harm	Total N		N in each group	Proportion (%)
NSSH: No	At least have data on NSSH or SSH: 9,295	NSSH only: 9,279	7,246	78.09
NSSH: Yes, 1–2 times			970	10.45
NSSH: Yes, 3–5 times			302	3.25
NSSH: Yes, 6–10 times			197	2.12
NSSH: Yes, > 10 times			564	6.08
SSH: No		SSH only: 9,278	8,285	89.30
SSH: Yes, 1–2 times			658	7.09
SSH: Yes, 3–5 times			152	1.64
SSH: Yes, 6–10 times			62	0.67
SSH: Yes, > 10 times			121	1.30
Mean age (Years)	22.3			

_NSSH=Non−suicidal self−harm; SSH=Suicidal self−harm; N = sample size_

We used the structural equation modelling R package OpenMx (Boker et al. 2011) to specify MR-DoC models. In each MR-DoC model, the instrumental variables (i.e., PRS of the corresponding predictor variable) were standardised to have a mean = 0 and standard deviation = 1. The exposures were treated as continuous variables, and adjusted for age and sex by individually regressing each variable on both covariates, and using the residuals in subsequent analyses. The residuals of these continuous variables were log-transformed to normalise the distributions for compatibility with parametric methods. The outcomes were ordinal variables, hence liability threshold models (Falconer 1965) were specified for them, in which the categories of the ordinal variables were regarded as expressions of underlying continuous and normally distributed liabilities for NSSH and SSH. For NSSH and SSH, four thresholds were estimated, corresponding to the five levels on the ordinal scales. For ordinal variables, sex and age were included as covariates in the threshold equations.

We assessed statistical significance of the estimates using 95% confidence intervals (CIs), which were computed using the full information maximum likelihood estimation. Model comparison was done by means of chi-squared testing and inspecting AIC values.

## Results

### Descriptive statistics

Table 1 shows the descriptive statistics for the prevalence of NSSH and SSH in the current study sample. Overall, NSSH has a prevalence of 21.9%, whereas SSH has a prevalence of 10.7%. This is comparable with the prevalence of lifetime NSSH (17.5%) and SSH (9.0%) in the 16–24 years age group in the England Adult Psychiatric Morbidity Survey (McManus et al. 2019). NSSH and SSH were highly correlated, with a polychoric correlation of *r* = 0.87, *p* < 0.001. However, this high correlation is likely to be mainly driven by the fact that most of those who had engaged in SSH had also engaged in NSSH (915/(915 + 72) = 92.7%), as shown in Figure S1. Descriptive statistics of the mental health measures are in Table S2.

### PRS regression analyses

Table 2 shows the results from PRS regression analyses. Out of 10 regression models run, after FDR correction, PRS for MDD significantly predicted child-rated MFQ and parent-rated MFQ (*q* = 3.61E-06 and 6.16E-05, respectively) and the PRS for ADHD significantly predicted parent-rated CPRS (*q* = 2.53E-15). PRS for schizophrenia did not predict any psychotic experiences subscales significantly after FDR correction. Similarly, the PRS for insomnia was not significantly associated with insomnia measured in TEDS.

**Table 2:** PRS regression analyses results

PRS	Predicted exposure	beta coefficient (95% CI)	p-value	q-value	R^2^	F-statistic
**MDD**	**Child-rated MFQ**	**0.277 (0.167,0.386)**	**7.22E-07**	**3.61E-06**	**0.004**	**24.29**
	**Parent-rated MFQ**	**0.137 (0.074,0.199)**	**1.85E-05**	**6.16E-05**	**0.004**	**22.64**
**ADHD**	**Parent-rated CPRS**	**0.771 (0.586,0.955)**	**2.53E-16**	**2.53E-15**	**0.011**	**60.08**
SCZ	Paranoia	-0.033 (-0.312,0.246)	0.817	0.908	-4.38E-06	0.07
	Hallucinations	0.009 (-0.149,0.167)	0.912	0.912	6.79E-06	0.16
	Cognitive disorganisation	0.075 (0.000,0.150)	0.051	0.102	0.001	4.55
	Grandiosity	0.074 (-0.042,0.190)	0.212	0.303	2.46E-04	0.59
	Anhedonia	-0.221 (-0.418,-0.024)	0.028	0.069	0.001	6.25
	Negative symptoms	0.075 (-0.030,0.180)	0.164	0.273	4.60E-04	2.00
Insomnia	Insomnia severity	0.028 (-0.099,0.154)	0.667	0.833	8.12E-05	0.28

_Note. PRS = polygenic risk score; MDD = Major depressive disorder; MFQ = Moods and Feelings Questionnaire; ADHD = Attention−deficit hyperactivity disorder; CPRS = Conner’s Parent Rating Scale; SCZ = schizophrenia. F−values were derived using lm() function. Bolded estimates are statistically significant with *q* < 0.05_

### MR-DoC models

Based on the results from PRS analyses, child-rated MFQ, parent-rated MFQ and parent-rated CPRS were included as exposures in MR-DoC models, with NSSH and SSH as the outcomes. With three exposures and two outcomes, six MR-DoC models were fitted in total. The estimated coefficients for the b1, b2 and g1 paths for these MR-DoC models are presented in Table 3.

**Table 3:** Estimates for the path coefficients and breakdown of phenotypic correlations between exposures and outcomes from MR-DoC models with their 95% confidence intervals (CIs)

Exposure	Outcome	b1 (instrumental path)	b2 (unmediated pleiotropy)	Phenotypic correlation (RPh)	Breakdown of RPh		
					RPh explained by A	RPh due to causal effect (g1)	RPh due to the polygenic risk score
MDD: Child- rated MFQ	NSSH	**0.066 (0.042,0.090)**	**0.087 (0.050,0.124)**	**0.370 (0.342,0.396)**	**0.170 (0.108,0.231)**	**0.194 (0.131,0.257)**	**0.006 (0.003,0.010)**
	SSH	**0.066 (0.041,0.090)**	**0.099 (0.052,0.144)**	**0.391 (0.357,0.423)**	**0.174 (0.092,0.255)**	**0.210 (0.125,0.295)**	**0.006 (0.003,0.011)**
ADHD: Parent- rated CPRS	NSSH	**0.101 (0.076,0.126)**	0.041 (-0.001,0.083)	**0.115 (0.078,0.152)**	-0.016 (-0.174,0.142)	0.127 (-0.032,0.285)	0.004 (0.000,0.009)
	SSH	**0.101 (0.076,0.126)**	**0.079 (0.027,0.131)**	**0.164 (0.118,0.209)**	-0.031 (-0.245,0.182)	0.187 (-0.028,0.402)	**0.008 (0.003,0.014)**
MDD: Parent- rated MFQ	NSSH	**0.064 (0.039,0.089)**	**0.093 (0.054,0.131)**	**0.194 (0.163,0.225)**	**0.097 (0.008,0.185)**	**0.092 (0.004,0.181)**	**0.006 (0.003,0.010)**
	SSH	**0.064 (0.039,0.089)**	**0.097 (0.050,0.144)**	**0.240 (0.204,0.274)**	0.068 (-0.047,0.181)	**0.165 (0.051,0.281)**	**0.006 (0.003,0.011)**

_NSSH=Non−suicidal self−harm; SSH=Suicidal self−harm; CPRS=Conner’s Parent Rating Scale; MFQ=Moods and Feelings Questionnaire; A=Additive genetic influences. Bolded estimates have 95% CIs not overlapping with zero, hence are considered as statistically significant. Phenotypic correlations (RPh) between the exposures and outcomes can be broken down into components that were driven by additive genetic effects, the causal effects and the polygenic risk scores._

**Fig. 3:**
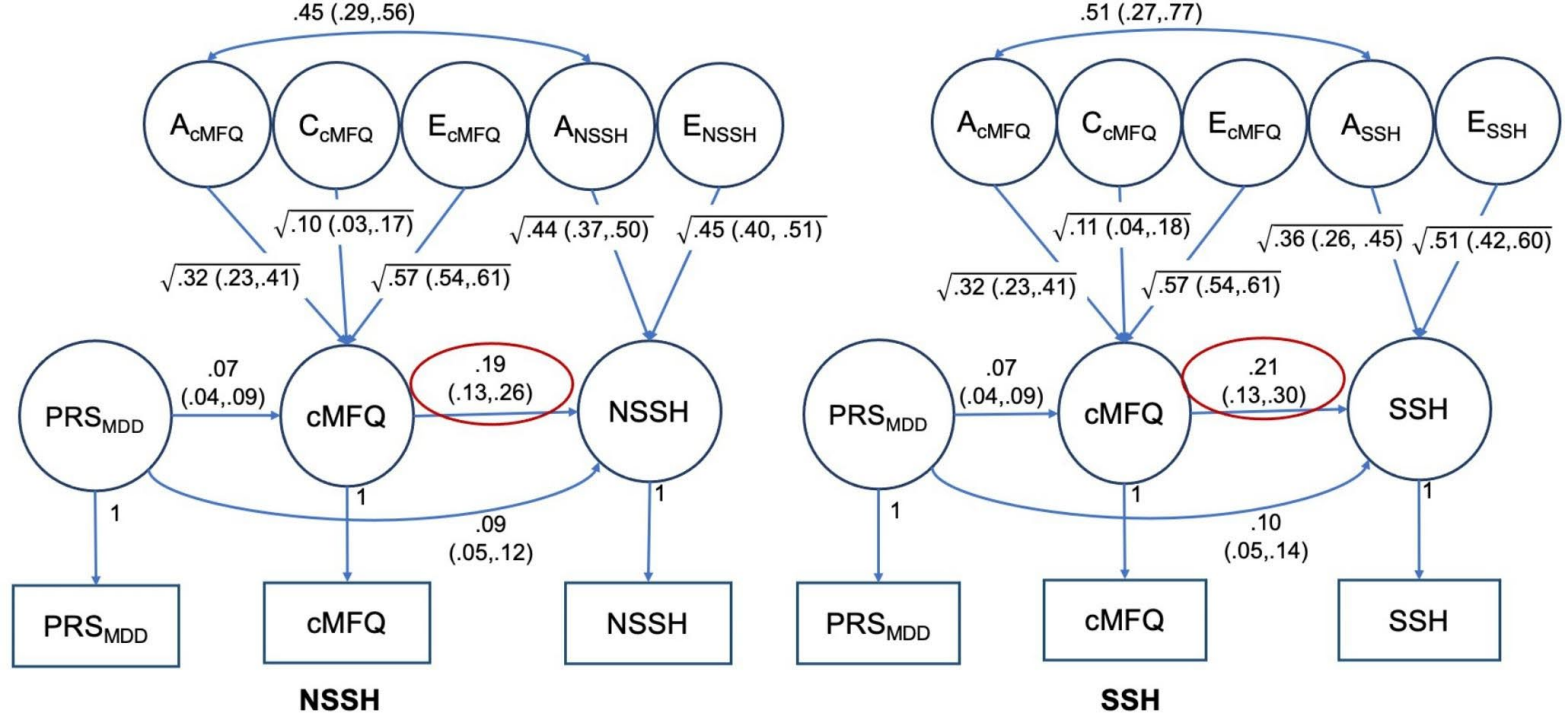
MR-DoC models with child-rated Moods and Feelings Questionnaire (cMFQ) as exposure, and non-suicidal and suicidal self-harm (NSSH and SSH respectively) as outcomes. MDD = major depressive disorder, PRS = polygenic risk score. Both shared and non-shared correlations (rC and rE) are constrained to zero. Red ovals indicate significance of the causal paths (g1)

**Fig. 4:**
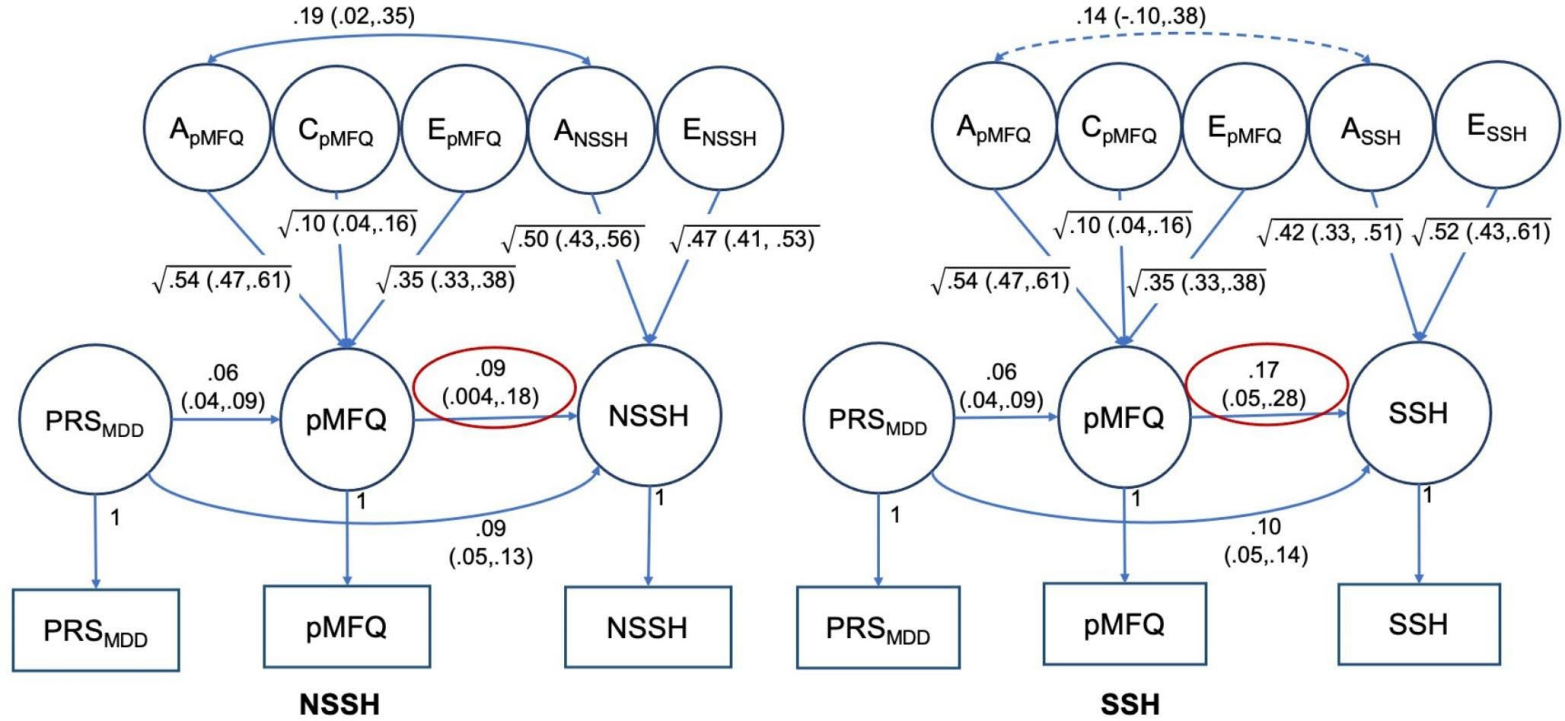
MR-DoC models with parent-rated Moods and Feelings Questionnaire (pMFQ) as exposure, and non-suicidal and suicidal self-harm (NSSH and SSH respectively) as outcomes. MDD = major depressive disorder, PRS = polygenic risk score. Both shared and non-shared correlations (rC and rE) are constrained to zero. Red ovals indicate significance of the causal paths (g1) Declarations

From the six MR-DoC models fitted, four of them showed significant causal effects from exposure to outcomes. As shown in Fig. 3, child-rated MFQ had significant causal effects on both NSSH (g1 = 0.194, 95% CIs: 0.131, 0.257) and SSH (g1 = 0.210, 95% CIs: 0.125, 0.295). Parent-rated MFQ also showed similar patterns of results but with attenuated causal effect sizes for both NSSH (g1 = 0.092, 95% CIs: 0.004, 0.181) and SSH (g1 = 0.165, 95% CIs: 0.051, 0.281), as shown in Fig. 4. For parent-rated CPRS, no significant causal effects were found.

The MR-DoC models also estimated unmediated pleiotropy paths. In all four models with MDD PRS as an instrumental variable, the unmediated pleiotropy path estimates (b2) were significant, as shown in Figs. 3 and 4. When ADHD PRS was used as the instrumental variable for parent-rated CPRS in two MR-DoC models, only the CPRS-SSH model had a significant unmediated pleiotropy path estimate (b2 = 0.079, 95% CI: 0.027,0.131) but not the CPRS-NSSH model.

### Sensitivity analyses

As suggested by Minică et al. (2018), we fixed rEs to various values to gauge the sensitivity of the causal path estimates (g1) to changes in the values of rE. Table S3 shows the values of g1 estimated with rE fixed at different values, starting from 0 to 0.25 at an increment of 0.05. As rE increased, the magnitude of g1 coefficients decreased and became not significant (when rE = 0.15 for child-rated MFQ models and when rE = 0.05 for parent-rated MFQ models, see Table S3). However, in all scenarios, the models in which the rEs were constrained to zero had the lowest AIC values, suggesting that these models may provide a better fit to the data, and that fixing rE to zero does not bias the causal path estimates.

## Discussion

Using the MR-DoC design, this study aimed to investigate causal relationships between self-harm and four mental health problems (MDD, ADHD, schizophrenia and insomnia) and self-harm, as well as aetiological differences between NSSH and SSH. We found that both parent-rated and child-rated depressive symptoms had direct causal relationships with both NSSH and SSH, suggesting there is no aetiological difference between NSSH and SSH in the context of their relationships with depressive symptoms. For ADHD, we did not find evidence of causal effects from parent-rated ADHD symptoms to either NSSH or SSH. PRS for schizophrenia and insomnia were not significantly associated with psychotic symptoms and insomnia severity respectively in the twin sample, hence they were not included in subsequent MR-DoC models to avoid violation of the first MR assumption.

### Causal pathways

In this study, depressive symptoms measured in adolescence appear to be causally related to both lifetime NSSH and SSH measured in early adulthood. The cognitive symptoms of depression may cause one to be more likely to self-harm, as posited by Wenzel and Beck (2008) that they may activate cognitive processes related to self-harm. Indeed, depressive symptoms were found to be influencing suicidal ideation through the cognitive triad (negative views of the self, the future and the world), which is the central feature of depressive symptoms (Alford et al. 1997; H.-J. Chang et al. 2007). The Interpersonal Theory of Suicide also posits that mental disorders such as depression may increase one’s risk of developing social isolation, feelings of loneliness and perception of burdensomeness, eventually increasing the risk towards suicide (Van Orden et al. 2010).

Although ADHD was found to be a significant causal risk factor for self-harm using standard MR (Lim et al. 2020), its putative causal effect was not found in the current study. A potential explanation for this may be because in the previous MR study (Lim et al. 2020), the genetic variants used reflect long-term exposures (e.g. long-term increased liability to ADHD and self-harm), whereas in the current study, the causal effects were estimated using ADHD symptoms rated at adolescence and lifetime self-harm measures collected at early adulthood. It is possible that the full-blown, lifetime causal effect of ADHD on self-harm is not reflected in the current MR-DoC model.

In the current study, although not statistically significant, the magnitudes of the causal path coefficients in the ADHD MR-DoC models are comparable with those with both child-rated or parent-rated depressive symptoms as exposures. Although the 95% CIs included zero, the ranges are wide and they skewed away from the null, suggesting there might be a lack of statistical power in the ADHD MR-DoC models to detect significance for the causal effect. Hence, the possibility of the causal effect of ADHD towards self-harm cannot be discarded.

### Aetiological similarities between NSSH and SSH

The causal estimates from both parent- and child-rated depressive symptoms to both NSSH and SSH were significant, whereas the causal estimates from parent-rated ADHD symptoms to both NSSH and SSH were not significant. This pattern of results suggests both NSSH and SSH are affected by the same exposures. There are suggestions that self-harm is probably best viewed as a continuum, with NSSH and SSH at different points of the continuum (Stanley et al. 1992). Although the current study does not directly show that NSSH and SSH are on the same continuum, the results suggest that they are aetiologically similar.

Nonetheless, across both child- and parent-rated depressive symptoms, their estimated causal effects on self-harm were always larger for SSH than for NSSH, suggesting having depressive symptoms may have larger impacts on SSH. However, these differences in magnitudes are not statistically significant as the 95% CIs were overlapping. Hence, these results should be interpreted with caution and there is no strong evidence to suggest that NSSH and SSH are aetiologically different.

### Unmediated pleiotropy pathways

One of the strengths of the MR-DoC design is it allows the direct testing of unmediated pleiotropy pathways. By leveraging this strength, we found that PRS for MDD had significant unmediated pleiotropy with both NSSH and SSH. For ADHD, the unmediated pleiotropy pathway was significant for SSH but not for NSSH, which may be due to lack of statistical power. The significance of unmediated pleiotropy for MDD PRS to both types of self-harm is also consistent with what was found in a previous study (Lim et al. 2020). MR-Egger regression is an MR sensitivity analysis used to detect unmediated pleiotropy (Bowden et al. 2015). In Lim et al. (2020), using MR-Egger regression, it was found that there is a significant unmediated pleiotropic pathway between depression and self-harm. This was replicated in the current study using the MR-DoC design.

By using MR-DoC design, we were able to test for the presence of causal effect and unmediated pleiotropy at the same time. We found that even with the presence of unmediated pleiotropy, causal effects from both parent-rated and child-rated depressive symptoms were found to be flowing to both NSSH and SSH. A potential explanation for the presence of unmediated pleiotropy is that one of the diagnostic criteria for MDD is having suicidal ideation (American Psychiatric Association 2013). Hence, the PRS for MDD may index genetic predisposition towards self-harm which does not necessarily have to be mediated through depressive symptoms. However, our results indicate depressive symptoms as an independent causal factor for self-harm after controlling for unmediated pleiotropic genetic influences. This also suggests that future genetically informed studies should ensure that measurement issues and unmediated pleiotropic pathways are accounted for when looking at the relationships between MDD and self-harm. Furthermore, the effect sizes of unmediated pleiotropic pathways between the MDD PRS and NSSH/SSH are larger than the direct pathway from the MDD PRS to depressive symptoms (although the differences in effect sizes are not statistically significant). This is surprising as we would expect the PRS for MDD to better predict depressive symptoms than it directly predicts self-harm. Several reasons can contribute to this result in the present sample. First, as mentioned above, given how MDD is measured, the PRS for MDD may include genetic predisposition towards self-harm. Second, in TEDS, self-harm was assessed in adulthood as a lifetime measure; depressive symptoms referred to experiences in the last two weeks during adolescence. Compared to the measure of self-harm, the measure of adolescent depression may thus suffer from greater measurement error and a greater discrepancy in age with the assessment of MDD, thus diluting the correlation with the MDD PRS. Studies using lifetime measures in adult samples for both depression and self-harm would be useful to address this question. In addition, a GWAS of depression excluding items referring to self-harm would be useful to avoid artificial pleiotropy arising from measurement issues.

### Research implications

The current study suggests that twin samples with genotyped data can be exploited to simultaneously examine the presence of unmediated pleiotropy and causal effects, providing a useful tool for causal inference. The current study also suggests that NSSH and SSH are aetiologically similar in terms of their causal relationships with MDD and ADHD. In DSM-5, NSSH and SSH were suggested as two separate conditions for further study (American Psychiatric Association 2013). We could not find evidence that major psychiatric disorders have a different impact on SSH versus NSSH. Our aetiological findings thus do not support the distinction between SSH and NSSH into separate conditions.

Using the MR-DoC model, causal effects were found from MDD to both NSSH and SSH, even after accounting for unmediated pleiotropy and shared additive genetic influences, with an assumption that there are no shared unique environmental factors, which will be addressed in the limitation section. This suggests that interventions designed for MDD may be helpful in addressing self-harm as well. For example, ketamine, which was found to have antidepressant effects, may also reduce suicide risks (Matveychuk et al. 2020).

The possibility of a causal effect from ADHD symptoms to self-harm should not be discarded as the non-significant causal estimate may be due to lack of statistical power. Indeed, it has been suggested that ADHD medication is associated with reduced suicide risk (Chang et al. 2020; Shoval et al. 2021). This demands a larger sample size of genotyped twins for future research.

### Strengths and limitations

One of the strengths in this study is the integration of the PRS as an instrumental variable and the DoC design. While the DoC model can be used on its own to estimate the causal effect of mental health problems on self-harm, the addition of a PRS allowed us to dissect the effect of polygenic scores for psychiatric disorders on self-harm into mediated and unmediated pleiotropic effects.

However, to ensure better resolution of the MR-DoC models, we fixed all rE environmental correlations to be zero, assuming that all non-shared environmental factors shared by exposures and outcomes pass through the causal pathway (g1). Although sensitivity analyses suggested that models with rEs fixed to zero (compared to models fixed at different rE values) have the best fit, in interpreting the causal relationships in this study, it is important to note that it assumes that non-shared environmental confounding is null. This is a strong assumption, as it is possible that there are other non-shared environmental factors which may contribute to the aetiology of depressive symptoms and self-harm, given that they are complex traits. For example, using the MZ twin differences design, two separate studies respectively showed that bullying victimisation is associated with self-harm (O’Reilly et al. 2021) and depressive symptoms (Singham et al. 2017). Note that such findings do not necessarily imply confounding, as non-shared environmental factors such as bullying may first affect mental health, which in turn, affects self-harm (i.e., bullying is not a confounder but its effect on self-harm is totally mediated by its effect on mental health). Nevertheless, results presented here should be interpreted cautiously with this assumption in mind. In addition to that, as recommended by Minică et al. (2018), alternative research designs and analytical approaches should also be used to replicate findings from MR-DoC analyses.

Although we were able to leverage the data from twins with their genotyped data, not all twins in this study were genotyped. This became a limitation of the study since there were missing PRS data. The PRS used were also not as powerful as the simulated PRS used in the original method paper by Minică et al. (2018). The largest amount of variance predicted among the instrumental paths was 1.1% for ADHD symptoms by PRS of ADHD, which was minuscule although comparable to variances predicted in other ADHD PRS studies, which can be as low as 0.7% (Ronald et al. 2021).

The current study used full information maximum likelihood to handle missing data, which assumes that the missing data were either missing completely at random (MCAR) or missing at random (MAR). One potential limitation is if the data are missing not at random (MNAR), this would have resulted in biased estimates (Newman 2014).

This study used self-harm measures which are ordinal variables with multiple levels, instead of using binary variables with “yes” or “no” responses, hence yielding a higher statistical power. We also used both parent and child-rated measures whenever possible. Despite these, it is difficult to interpret the estimates derived from this study as the continuous exposures were log-transformed and the ordinal outcomes were assumed to each have an underlying continuous and normally distributed liability.

In this study, whenever possible, both parent- and child-rated measures were included for each mental health problem so that the causal effects estimated from different raters can be compared. However, this was not possible for ADHD as we only had access to parent-rated ADHD symptoms, which is a limitation of this study.

## Conclusion

In conclusion, by assuming that there are no non-shared environmental confounds, we found that there are significant causal effects from both parent-rated and child-rated depressive symptoms to both NSSH and SSH, despite the presence of unmediated pleiotropy. The results showed that the causal effects of risk factors for NSSH and SSH are similar, suggesting no aetiological difference between them.

## Electronic Supplementary Material

Below is the link to the electronic supplementary material.

Supplementary Material 1

## Data Availability

Data for this study came from the Twins Early Development Study (TEDS). Researchers can apply for access to the data: https://www.teds.ac.uk/researchers/teds-data-access-policy.

## Code Availability

The codes used for analysis are available at: https://kai-lim.github.io/MR-DoC-self-harm/.
