# Correction to: Investigating the Causal risk Factors for self-harm by Integrating Mendelian Randomisation Within twin Modelling

**DOI:** 10.1007/s10519-022-10117-8

**Published:** 2022-10-11

**Authors:** Kai Xiang Lim, Olakunle Ayokunmi Oginni, Kaili Rimfeld, Jean-Baptiste Pingault, Frühling Rijsdijk

**Affiliations:** 1 Social, Genetic and Developmental Psychiatry Centre, Institute of Psychiatry, Psychology and Neuroscience, King’s College London, London, UK; 2 Department of Psychology, Royal Holloway University of London, London, UK; 3 Department of Clinical, Educational and Health Psychology, Division of Psychology and Language Sciences, University College London, London, UK; 4 Faculty of Social Sciences, Anton de Kom University of Suriname, Paramaribo, Suriname

**Correction to: Behavior Genetics.**

10.1007/s10519-022-10114-x.

In the original version of this article, unfortunately the article note was missed. The article note should be displayed as “Dr. Jean-Baptiste Pingault and Dr. Frühling Rijsdijk are shared senior authors”. This has been corrected by publishing this correction article. The original article has been corrected.

