# Supplementary material for: Investigating the causal risk factors for self-harm by integrating Mendelian randomisation within twin modelling
Source: Behav Genet. 2022 Sep 14;52(6):324–37. doi: 10.1007/s10519-022-10114-x (PMC9646538; doi:10.1007/s10519-022-10114-x)
Supplement: Supplementary file 1 — Supplementary Material 1 [file 10519_2022_10114_MOESM1_ESM.docx]

### **Investigating the aetiology of self-harm by integrating Mendelian randomisation within twin modelling**

### **Supplementary Information**

Kai Xiang Lim^1^, Olakunle Ayokunmi Oginni^1^, Kaili Rimfeld^1,2^, Jean-Baptiste Pingault^1,3^*, Frühling Rijsdijk^1,4^*

^1^Social, Genetic and Developmental Psychiatry Centre, Institute of Psychiatry, Psychology and Neuroscience, King’s College London, London, UK.

^2^Department of Psychology, Royal Holloway University of London, London, UK.

^3^Department of Clinical, Educational and Health Psychology, Division of Psychology and Language Sciences, University College London, London, UK.

^4^Faculty of Social Sciences, Anton de Kom University of Suriname, Paramaribo, Suriname.

Table S1. ACE/AE estimates for exposures and outcomes from MR-DoC models (95% confidence intervals)

| Exposure | Outcome | Exposures | | | Outcomes | |
| --- | --- | --- | --- | --- | --- | --- |
|  |  | A (95% CI) | C (95% CI) | E (95% CI) | A (95% CI) | E (95% CI) |
| Child-rated MFQ | NSSH | 0.322  (0.229,0.413) | 0.103  (0.032,0.174) | 0.571  (0.537,0.606) | 0.435  (0.372,0.495) | 0.452  (0.396,0.512) |
|  | SSH | 0.317  (0.225,0.409) | 0.108  (0.036,0.178) | 0.570  (0.536,0.606) | 0.361  (0.264,0.452) | 0.510  (0.424,0.603) |
| Parent-rated CPRS | NSSH | 0.722  (0.670,0.776) | 0.111  (0.059,0.161) | 0.157  (0.146,0.168) | 0.523  (0.462,0.583) | 0.462  (0.405,0.523) |
|  | SSH | 0.722  (0.670,0.776) | 0.112  (0.059,0.162) | 0.156  (0.146,0.168) | 0.452  (0.358,0.549) | 0.515  (0.430,0.608) |
| Parent-rated MFQ | NSSH | 0.543  (0.472,0.614) | 0.102  (0.042,0.161) | 0.351  (0.328,0.375) | 0.498  (0.434,0.558) | 0.466  (0.409,0.528) |
|  | SSH | 0.542  (0.471,0.613) | 0.104  (0.043,0.162) | 0.350  (0.327,0.375) | 0.421  (0.326,0.509) | 0.518  (0.432,0.612) |

_Note. MDD = Major depressive disorder; MFQ = Moods and Feelings Questionnaire; CPRS = Conner’s Parent Rating Scale_

Figure S1. Number and percentages of participants who reported NSSH and SSH.


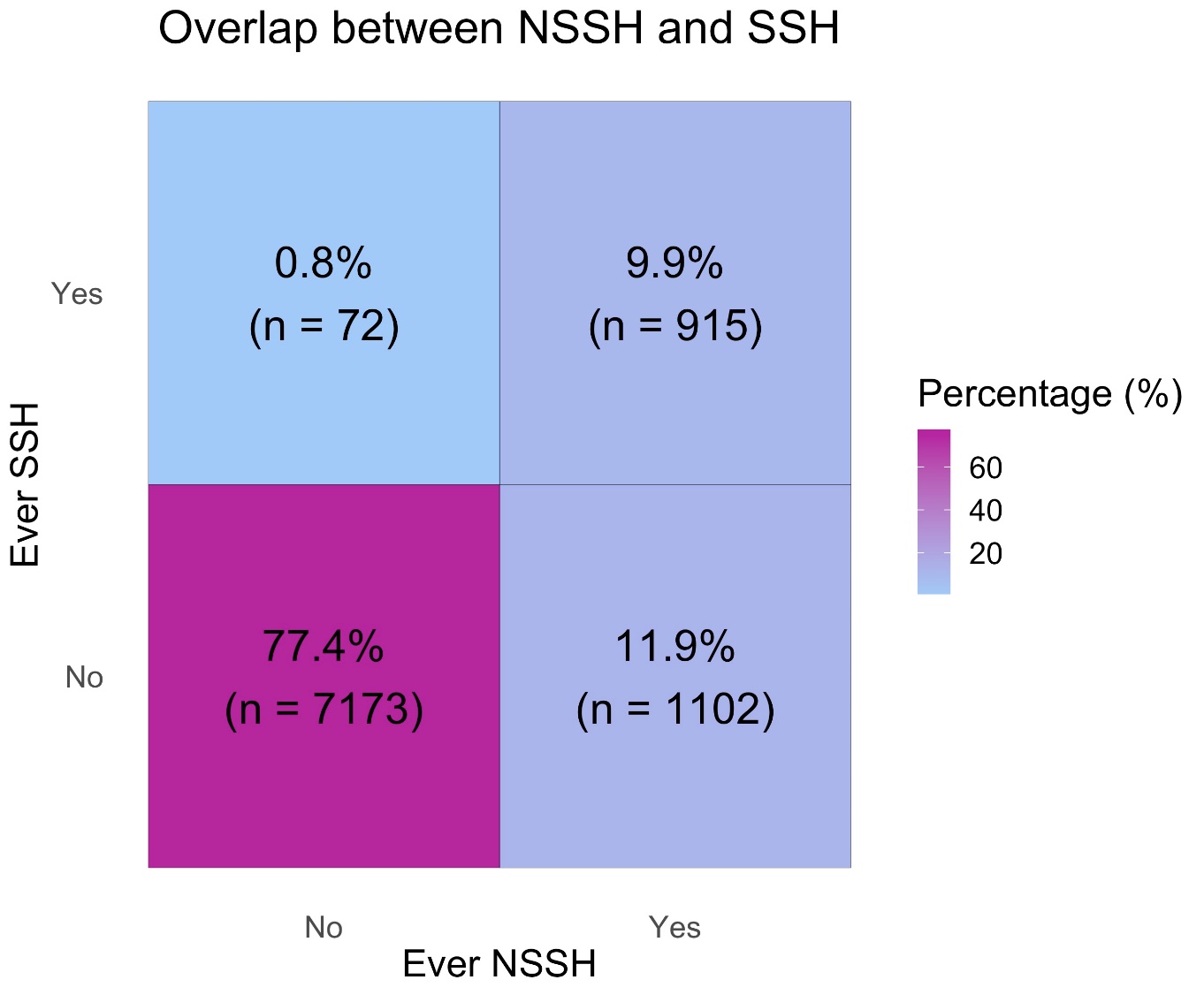


^Note. Participants included in analyses are those who at least have data on either NSSH or SSH. Hence, there were participants who have responded whether they had ever NSSH but have missing data for SSH-related questions, and vice versa. Partially missing data (e.g., participants who endorsed NSSH question but not SSH question) are not included in this figure. In total, there are 33 participants who have partially missing data.^

Table S2. Descriptive statistics for mental health measures of the sample used in analyses

| Measure | Sample size (N) | Mean | Minimum | Maximum | SD |
| --- | --- | --- | --- | --- | --- |
| child-rated MFQ | 10,141 | 3.64 | 0 | 26 | 4.44 |
| parent-rated MFQ | 10,161 | 1.00 | 0 | 22 | 2.32 |
| parent-rated CPRS | 10,157 | 6.89 | 0 | 53 | 7.55 |
| child-rated paranoia | 10,132 | 12.12 | 0 | 72 | 10.66 |
| child-rated hallucinations | 10,141 | 4.71 | 0 | 45 | 6.12 |
| child-rated cognitive disorganisation | 10,128 | 3.99 | 0 | 11 | 2.87 |
| child-rated grandiosity | 10,133 | 5.37 | 0 | 24 | 4.49 |
| child-rated anhedonia | 10,136 | 16.27 | 0 | 50 | 7.87 |
| parent-rated negative symptoms | 10,157 | 2.93 | 0 | 30 | 3.97 |
| child-rated insomnia severity | 7,852 | 3.85 | 0 | 28 | 4.22 |

Table S3. Estimated causal effects (g1) when rE is fixed at different values.

| Exposure | Outcome | Value of fixed rE | AIC | Estimated g1 (95%CI) | rE freely estimated when g1 fixed to zero |
| --- | --- | --- | --- | --- | --- |
| Child-rated MFQ | NSSH | **0** | **63865.96** | **0.194 (0.131, 0.257)** | 0.203 |
|  |  | **0.05** | **63868.21** | **0.148 (0.084, 0.211)** |  |
|  |  | **0.1** | **63868.49** | **0.102 (0.038, 0.165)** |  |
|  |  | 0.15 | 63868.79 | 0.054 (-0.010, 0.119) |  |
|  |  | 0.2 | 63869.10 | 0.006 (-0.059, 0.071) |  |
|  |  | 0.25 | 63869.44 | -0.043 (-0.110, 0.022) |  |
|  | SSH | **0** | **57845.23** | **0.210 (0.125, 0.295)** | 0.206 |
|  |  | **0.05** | **57847.30** | **0.161 (0.075, 0.246)** |  |
|  |  | **0.1** | **57847.40** | **0.111 (0.025, 0.197)** |  |
|  |  | 0.15 | 57847.51 | 0.061 (-0.027, 0.147) |  |
|  |  | 0.2 | 57847.65 | 0.009 (-0.080, 0.097) |  |
|  |  | 0.25 | 57847.81 | -0.044 (-0.135, 0.045) |  |
| Parent-rated MFQ | NSSH | **0** | **80198.68** | **0.092 (0.004, 0.181)** | 0.070 |
|  |  | 0.05 | 80201.07 | 0.032 (-0.057, 0.121) |  |
|  |  | 0.1 | 80201.51 | -0.028 (-0.117, 0.060) |  |
|  |  | 0.15 | 80201.97 | -0.090 (-0.179, 0.000) |  |
|  |  | 0.2 | 80202.48 | -0.152 (-0.243, -0.062) |  |
|  |  | 0.25 | 80203.02 | -0.217 (-0.309, -0.126) |  |
|  | SSH | **0** | **74019.07** | **0.165 (0.051, 0.281)** | 0.122 |
|  |  | 0.05 | 74021.34 | 0.102 (-0.013, 0.217) |  |
|  |  | 0.1 | 74021.65 | 0.038 (-0.078, 0.153) |  |
|  |  | 0.15 | 74021.99 | -0.027 (-0.144, 0.089) |  |
|  |  | 0.2 | 74022.37 | -0.094 (-0.212, 0.024) |  |
|  |  | 0.25 | 74022.78 | -0.162 (-0.282, -0.043) |  |
| Parent-rated CPRS | NSSH | 0 | 63413.84 | 0.127 (-0.032, 0.285) | 0.064 |
|  |  | 0.05 | 63416.08 | 0.039 (-0.121, 0.197) |  |
|  |  | 0.1 | 63416.48 | -0.050 (-0.210, 0.109) |  |
|  |  | 0.15 | 63417.03 | -0.141 (-0.302, 0.019) |  |
|  |  | 0.2 | 63417.72 | -0.233 (-0.395, -0.072) |  |
|  |  | 0.25 | 63418.54 | -0.329 (-0.493, -0.166) |  |
|  | SSH | 0 | 57226.27 | 0.187 (-0.028, 0.402) | 0.076 |
|  |  | 0.05 | 57228.85 | 0.093 (-0.122, 0.309) |  |
|  |  | 0.1 | 57229.53 | -0.002 (-0.218, 0.215) |  |
|  |  | 0.15 | 57230.30 | -0.098 (-0.316, 0.119) |  |
|  |  | 0.2 | 57231.16 | -0.197 (-0.417, 0.022) |  |
|  |  | 0.25 | 57232.10 | -0.300 (-0.522, -0.079) |  |

^Note. AIC = Akaike Information Criteria; rE = non-shared environmental correlation; g1 = causal effect in MR-DoC model; MFQ = Moods and Feelings Questionnaire; CPRS = Conner’s Parent Rating Scale; NSSH = non-suicdal self-harm; SSH = suicidal self-harm. Bolded estimates indicate statistical significance.^
